# Increases in Hydrophilicity and Charge on the Polar Face of Alyteserin 1c Helix Change its Selectivity towards Gram-Positive Bacteria

**DOI:** 10.3390/antibiotics8040238

**Published:** 2019-11-27

**Authors:** Yamil Liscano, Constain H. Salamanca, Lina Vargas, Stefania Cantor, Valentina Laverde-Rojas, José Oñate-Garzón

**Affiliations:** 1 Grupo de Génetica, Regeneración y Cáncer, Instituto de Química, Facultad de Ciencias Exactas y Naturales, Universidad de Antioquia, A.A., Medellín 1226, Colombia; yamil.liscano@udea.edu.co; 2 Laboratorio de Diseño y Formulación de Productos Químicos y Derivados, Departamento de Ciencias Farmacéuticas, Facultad de Ciencias Naturales, Universidad Icesi, Cali 760035, Colombia; chsalamanca@icesi.edu.co; 3 Grupo de Investigación en Química y Biotecnología (QUIBIO), Facultad de Ciencias Básicas, Universidad Santiago de Cali, Calle 5 No. 62-00, Cali 760035, Colombia; lina-vargas29@hotmail.com (L.V.); stefania.cantor00@usc.edu.co (S.C.); valentina.laverde00@usc.edu.co (V.L.-R.)

**Keywords:** Antimicrobial peptide, α-helix, membrane model, net charge, antimicrobial selectivity

## Abstract

Recently, resistance of pathogens towards conventional antibiotics has increased, representing a threat to public health globally. As part of the fight against this, studies on alternative antibiotics such as antimicrobial peptides have been performed, and it has been shown that their sequence and structure are closely related to their antimicrobial activity. Against this background, we here evaluated the antibacterial activity of two peptides developed by solid-phase synthesis, Alyteserin 1c (WT) and its mutant derivative (ΔM), which shows increased net charge and reduced hydrophobicity. These structural characteristics were modified as a result of amino acid substitutions on the polar face of the WT helix. The minimum inhibitory concentration (MIC) of both peptides was obtained in Gram-positive and Gram-negative bacteria. The results showed that the rational substitutions of the amino acids increased the activity in Gram-positive bacteria, especially against *Staphylococcus* *aureus*, for which the MIC was one-third of that for the WT analog. In contrast to the case for Gram-positive bacteria, these substitutions decreased activity against Gram-negative bacteria, especially in *Escherichia coli*, for which the MIC was eight-fold higher than that exhibited by the WT peptide. To understand this, models of the peptide behavior upon interacting with membranes of *E. coli* and *S. aureus* created using molecular dynamics were studied and it was determined that the helical stability of the peptide is indispensable for antimicrobial activity. The hydrogen bonds between the His20 of the peptides and the phospholipids of the membranes should modulate the selectivity associated with structural stability at the carboxy-terminal region of the peptides.

## 1. Introduction

Antimicrobial peptides (AMPs) are molecules produced via innate immunity, which are expressed by the host when challenged by an infectious agent [1]. These molecules generally exert their antimicrobial activity via interaction with the anionic membranes of the microorganisms, altering the phospholipid packaging while integrity and permeability are lost [2], thus killing the microorganism. For this reason, the development of resistance to AMPs is lower than that to conventional antibiotics [3], which are typically directed towards specific targets, especially proteins [4]. Thus, to acquire resistance toward AMPs, microbes would need to “redesign” the lipid composition of their membranes, but this cannot be done without damaging themselves [5]. Most AMPs that have been naturally isolated have a cationic charge between +2 and +9 at physiological pH, and consist of between 40% and 60% hydrophobic residues, conferring them with amphipathicity [6]. Net charge is an essential property for antimicrobial activity, since it is responsible for the initial interaction between the peptide and the anionic membranes of the pathogens, while hydrophobic residues contribute to the insertion of the peptide into the hydrophobic membrane core [7,8]. AMPs tend to acquire secondary structures of the α-helix type in an amphipathic environment such as that containing a solvent that simulates the amphipathicity of a membrane, 50% trifluoroethanol–water [9]. The α-helix is one of the most common structures in peptides, with hydrophobic residues on one side of the helix and cationic/hydrophilic residues on the other, and both sides of the helix being important for antibacterial activity [7].

A peptide of 23 amino acid residues with a net charge of +2, Alyerserin-1c (WT), was isolated for the first time from the cutaneous secretions of the *Alytes obstetricans* toad. After evaluating the biological activity, the results showed a selective inhibitory effect against Gram-negative bacteria and low hemolytic activity in human erythrocytes (HC_50_ = 220 µM) [10]. Furthermore, the three-dimensional structure of Alyteserin-1c was characterized as an extended α-helix between Leu2 and Val21 residues [9]. In a recent study by Aragón-Muriel et al. [11], it was reported that the Alyteserin-1c peptide and its more cationic and hydrophilic analog exhibited different secondary structures depending on the nature of the environment, including disordered structures and β-sheets. 

Each antimicrobial peptide has a particular way of acting on the microorganism membranes because its activity is closely related to its sequence, structure, and membrane composition. For this reason, it is difficult to understand the mechanism of action of each peptide reported in the literature. In this context, in recent years, peptide–membrane studies using molecular dynamics have helped to overcome this bottleneck and to understand the mechanism of action of several antimicrobial peptides [11,12,13,14], due to the detailed information, accuracy of the results, and reductions of study time and cost.

In this study, ΔM peptide with a net charge of +5 was designed from the WT peptide sequence, in which hydrophobic amino acids located on the polar face of the helix were replaced by hydrophilic amino acids, according to the suggestions of Bordo and Argos [15], increasing the amphipathicity and reducing the hydrophobicity. Both peptides were synthesized manually by solid-phase synthesis (SPPS), and their antimicrobial and hemolytic activities were reported. To determine the antimicrobial selectivity, diverse Gram-negative and Gram-positive strains were used, including *S. aureus* strains with variable responses of resistance to β-lactams. Moreover, in order to understand the change of bacterial selectivity, peptide–membrane simulations were performed by applying molecular dynamics (MD).

## 2. Results

### 2.1. Peptide Design and Sequence Characteristics

The WT peptide composed of 23 amino acid residues has on its polar face an anionic residue (E4), two hydrophobic residues (A8 and A18), and a neutral polar residue (S12). These residues were replaced by arginine, serine, lysine, and serine, respectively, in order to increase the charge, hydrophilicity, and amphipathicity [16].

### 2.2. Molecular Modeling of the Peptides and Quality Testing

**Figure 1:**
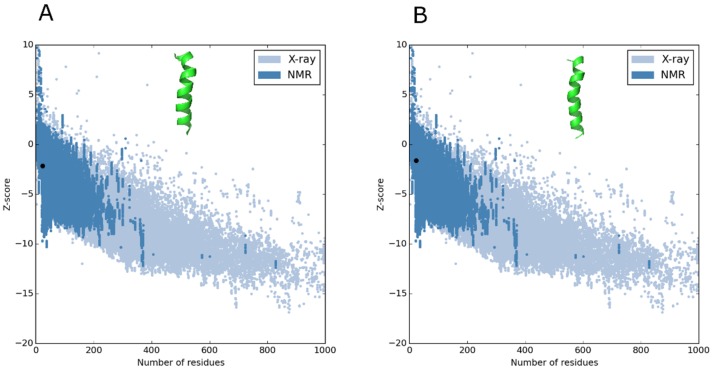
(**A**). Z-SCORE of the 3D structure model of the Alyteserin 1c (WT) peptide using the PROSA structure validation software. (**B**). Z-SCORE of the ΔM peptide 3D structure model using the PROSA structure validation software. Both figures illustrate the position of the structures modeled according to the number of black-shaped residues in the desired region of structures obtained from nuclear magnetic resonance (NMR) imaging.

To avoid mistakes in choosing the best peptide structural model, the tools PROSA and RAMPAGE can be used to refine and validate protein or peptide models. After processing the data, both peptides exhibited helical structures, which were within the viable quality ranges (Figure 1). Z-score values obtained by PROSA software, which assumes that most of the data points in a multidimensional NMR spectrum are at locations not occupied by signals [17], showed that both peptides were within the favorable region of structures, suggesting that they have characteristics of native structures [18].

**Figure 2:**
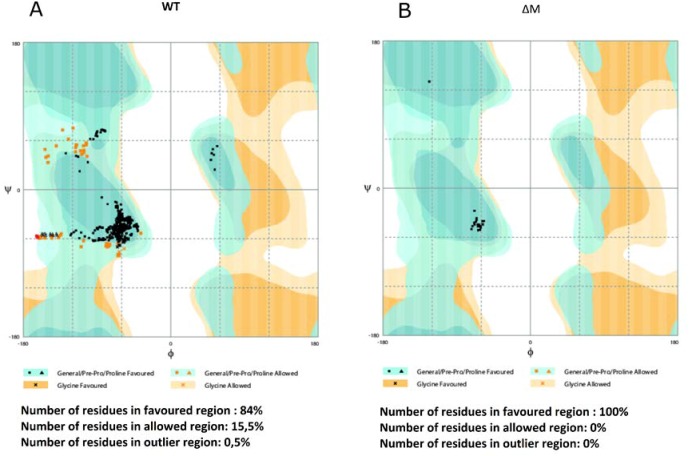
Ramachandran plot obtained from RAMPAGE software. Three types of region are displayed: favorable (blue), allowed (orange), and atypical regions (white). (**A**) WT peptide and (**B**) ∆M peptide.

Regarding the RAMPAGE validation (Figure 2), it was observed that the ∆M peptide had all the residues within the favorable region, suggesting high structural reliability. On the other hand, the WT peptide exhibited 84% of its residues within the favorable region and 65% in the permitted region, suggesting that the parameters of the peptide structure remain within the limits of good quality and stability [19].

### 2.3. Antibacterial Activity

By increasing the hydrophilicity and the charge on the polar face of the Alyteserin 1c helix, the antibacterial activity was increased only in Gram-positive bacteria, especially in *S. aureus* ATCC25923, for which the MIC was reduced from 250 to 65 μM, meaning three-fold higher antimicrobial activity than that exhibited by the WT peptide. β-lactam-resistant strains, *S. aureus* ATCC29213 (MSSA, ampicillin-resistant) and *S. aureus* ATCC43300 (MRSA), were only sensitive to the ΔM peptide at the highest evaluated concentration (Table 1). Likewise, in the other Gram-positive strains used to evaluate the antibacterial activity of the peptides, *L. monocytogenes* and *B. cereus*, once again the ΔM peptide showed greater selectivity towards these bacteria with MICs of 62.5 and 125 μM, respectively. This contrasted with the WT peptide that exhibited an MIC of 125 μM for *L. monocytogenes*, while in *B. cereus* no inhibition was observed at the maximum evaluated concentration (Table 1).

The antibacterial activity against Gram-negative bacteria was also determined and, in contrast to that observed in Gram-positive bacteria, the WT peptide had higher antibacterial activity than the ΔM peptide in all strains. The WT peptide exhibited MICs of 15.2, 31.3, and 62.5 μM for *E. coli, P. aeruginosa*, and *S. typhimurium*, respectively, while the MICs exhibited by the ΔM peptide were 62.5, 250, and 125 μM for each Gram-negative strain, respectively (Table 1).

### 2.4. Hemolytic Effect

**Figure 3:**
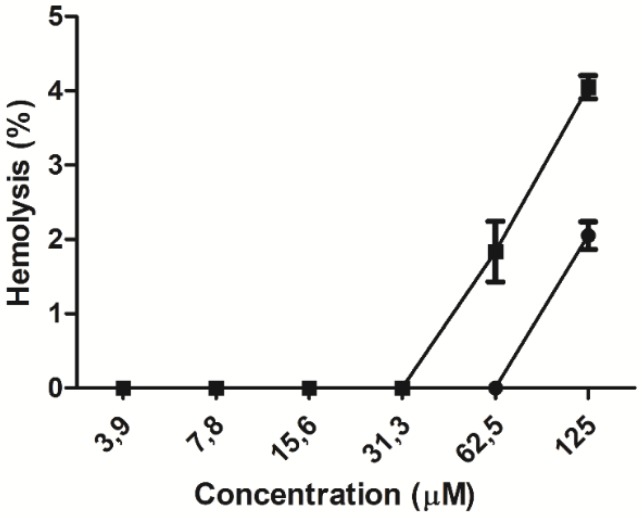
Percentage of hemolytic activity measured in human peripheral blood erythrocytes in the presence of WT (●) and ΔM (■) peptides.

The hemolytic activity of the peptides was evaluated in human erythrocytes. At the lowest peptide concentrations, ranging from 3.9 to 31.3 µM, no hemolytic effect was observed. At higher concentrations, a hemolytic concentration–response effect was observed for each peptide (Figure 3), lysing 2% and 4% of the total erythrocyte population at the maximum concentration evaluated for the WT and ΔM peptides, respectively. The minimum hemolytic concentration (MHC) was 62.5 µM for the WT peptide and 31.3 µM for the ΔM peptide (Table 1), suggesting that amino acid residue substitutions in the modified peptide double its hemolytic activity.

### 2.5. Prediction of Cleavage Sites of Staphylococcal Peptidase I

According to the results of the antibacterial activity, more WT peptide is needed than for its ΔM analog to inhibit the growth of *S. aureus* ATCC25923 (Table 1). In fact, the WT peptide showed no activity in both strains of *S. aureus* resistant to β-lactams (ATCC29213 and ATCC43300), even at concentrations as high as 250 µM (Table 1). To determine qualitatively whether the resistance is due in part to the proteases excreted by *S. aureus*, prediction of the sites cut in both peptides was performed. The results obtained with a peptide digestion tool (ExPASY peptide cutter) indicated that only the WT peptide is digested with the enzyme staphylococcal peptidase I (Table 2). Our analysis revealed that this peptide is cleaved at one site (E4), while the ΔM peptide remains unchanged.

### 2.6. Modeling the Interaction of Peptides with Membranes of E. coli and S. aureus

**Figure 4:**
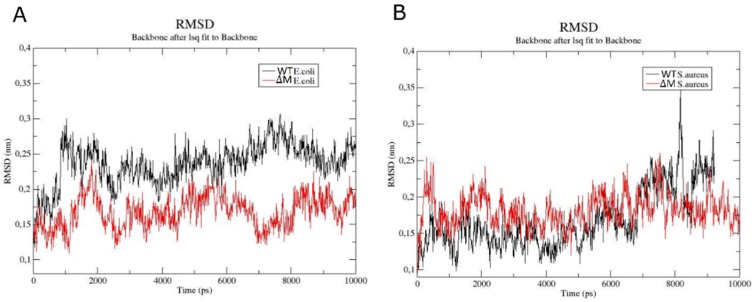
RMSD analysis of the backbone atoms of WT (black color) and ∆M (red color) peptides in *E. coli* (**A**) and *S. aureus* (**B**) membranes.

To try to understand the emergence of ∆M peptide selectivity towards Gram-positive bacteria, molecular dynamic studies were performed using membrane models. RMSD analysis of the backbone of the peptides for the *E. coli* membrane (Figure 4A) showed that the WT peptide was more stable since its structure showed deviations between 0.2 and 0.3 nm, meaning a variation of 0.1 nm, while the ∆M peptide had a range between 0.1 and 0.25 nm, with a variation of 0.15 nm, at 2000 ps (2 ns). On the other hand, the RMSD of the peptides in *S. aureus* membrane (Figure 4B) showed that both exhibited similar behavior; however, from 8000 ps (8 ns), the WT peptide was destabilized, reaching maximum variation of 0.25 nm, which was higher than the maximum variation exhibited by the ∆M peptide (0.1 nm).

**Figure 5:**
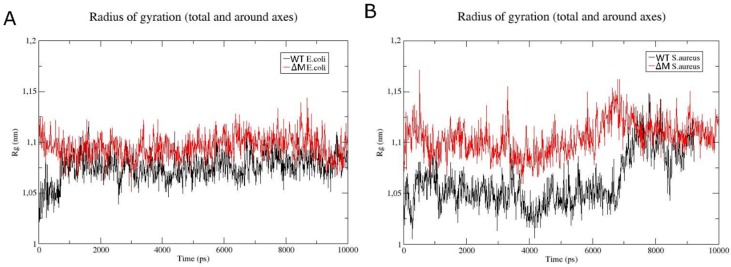
Analysis of radius of gyration of WT (black color) and ∆M (red color) peptides in *E. coli* (**A**) and *S. aureus* (**B**) membranes.

The radius of gyration is defined as the distribution of atoms of a protein around its axis and, specifically, is the length that represents the distance between the point when it is rotating and the point where the energy transfer has the maximum effect [20]. In addition, it serves as an indicator of protein structure compaction [21]. Figure 5 shows the radius of gyration of each peptide in the membranes of *E. coli* (Figure 5A) and *S. aureus* (Figure 5B). In membrane models that simulate Gram-negative bacteria, the WT peptide showed variation of the radius of gyration of 0.05 nm, which is very similar to that of ∆M; however, in the time interval of 8000–9000 ps, there is slight variation of approximately 0.06 nm in the ∆M. On the other hand, in the *S. aureus* system, there is a considerable change in the WT peptide of approximately 2 nm with respect to 1 nm in the ∆M (Figure 5B), originating from 7000 ps (7 ns).

**Figure 6:**
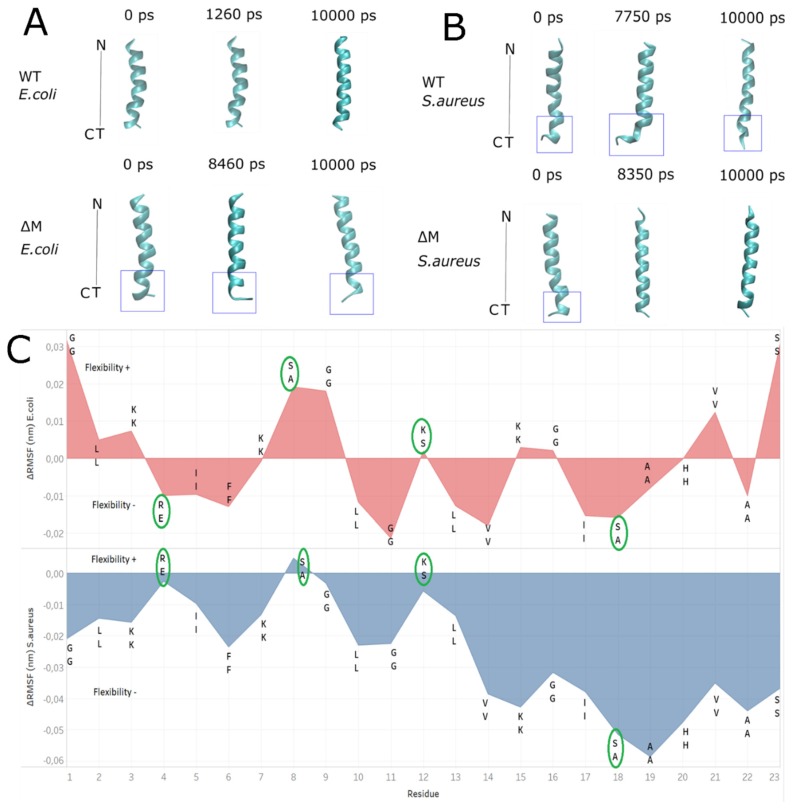
Comparison of the stability and flexibility of the helical structures of WT and ∆M peptides. (**A**) *E. coli* membrane model and (**B**) *S. aureus* membrane model. In blue is indicated the notorious changes in alpha helix structure. (**C**) ΔRMSF (nm) between WT and ∆M peptides, showing changes of residues in green circles. It is also showed positive values as flexibility + and negative values as flexibility -. The top letters are from sequence of ∆M peptide and the bottom letters belongs to sequence of WT peptide.

Alongside the analysis of stability of the peptides interacting with membrane models, changes in the conformation of the structures throughout the simulations were observed (Figure 6 and Supplementary Figure S1), where the WT peptide maintains its structure in *E. coli* membranes over time but becomes more unstable in *S. aureus* membranes at 7750 ps, specifically in the carboxy-terminal region including residues 18–23 (blue box, Figure 6B). Interestingly, the contrary effect is observed in ∆M peptide, where its helical structure is more stable in *S. aureus* than in *E. coli* membrane. In fact, the ∆M peptide undergoes slight instability, losing its helical structure between residues 19 and 22 between 8000 and 9000 ps (Supplementary Figure S1). Figure 6c shows the ∆RMSF between the ∆M and WT peptide. This plot allows to observe the flexibility of the residues of the ∆M peptide in both *E. coli* and *S. aureus* model membranes, detailing a greater stiffness in *S. aureus* with negative flexibility values except residue 8, where the change from alanine to serine slightly increased flexibility in the ∆M peptide. On the other hand, in *E. coli* the flexibility of the ∆M peptide was much greater than that in *S. aureus* model membrane, mainly in the amino and carboxyl terminal. Serine at position 8 of the ∆M peptide increased its flexibility again.

RMSD, ∆RMSF, radius of gyration values and secondary structure, suggest that there is a greater stability of the WT peptide in *E. coli* membrane than its analogue ∆M. On the contrary, in *S. aureus* membrane a decrease in the flexibility of the residues for ∆M was observed, contributing to the conservation of the helical structure, which is associated with experimental assays where the activity against Gram-positive bacteria was increased after residue substitutions (Table 1). 

**Figure 7:**
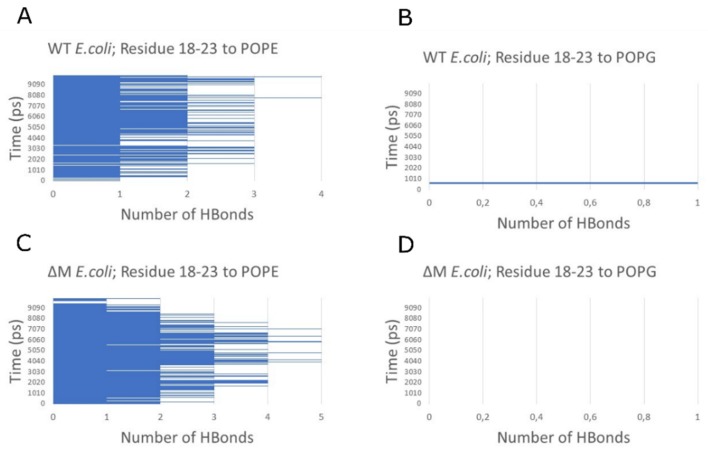
Hydrogen bond count between residues 18–23 of the peptides and phospholipids of *E. coli* membrane. WT interacting with POPE (**A**) and POPG (**B**). ∆M interacting with POPE (**C**) and POPG (**D**).

**Figure 8:**
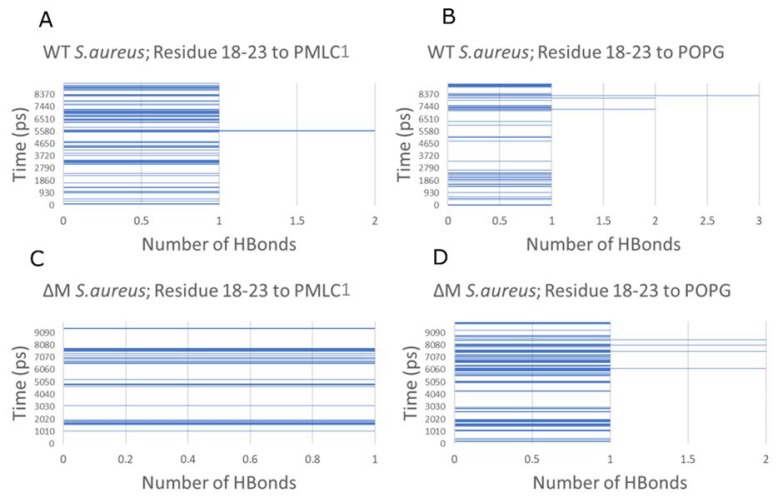
Hydrogen bond count between residues 18–23 of the peptides and phospholipids of the *S. aureus* membrane. WT interacting with PMLC1 (**A**) and POPG (**B**). ∆M interacting with PMLC1 (**C**) and POPG (**D**).

To explore whether the difference in phospholipid composition between Gram-negative and Gram-positive membranes influences the instability of the WT peptide, an analysis of the hydrogen bond count between the most unstable region of the peptides (residues 18–23) and POPE, POPG, and PMLC1 was performed. It was determined that there is a greater trend to form hydrogen bonds between peptides and POPE, a component of the *E. coli* membrane. This affinity promotes the constant formation of an average of two hydrogen bonds with POPE, while with POPG the interaction is almost nil (Figure 7). For *S. aureus*, the peptide–lipid affinity changes due to the hydrogen bonds being formed equally with POPG and PMLC1 (specifically, one hydrogen bond in each case) (Figure 8).

**Figure 9:**
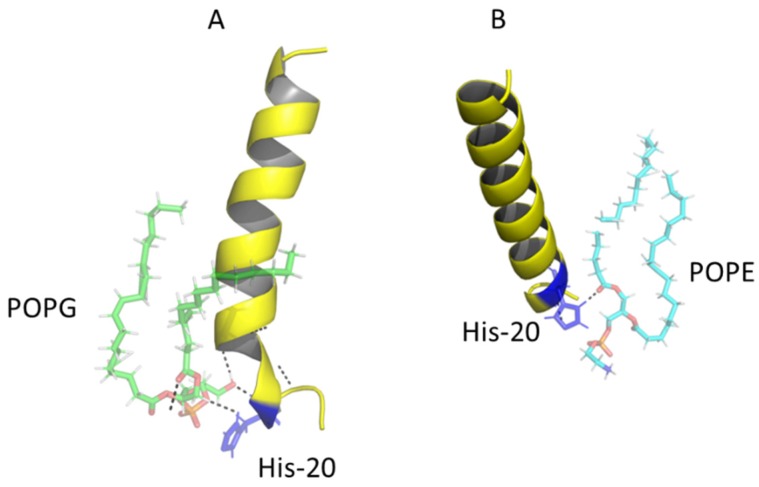
Hydrogen bonds between His20 residue of the WT peptide and membrane models of *S. aureus* (**A**) and *E. coli* (**B**).

Finally, a more detailed analysis of the simulation of the WT peptide with membrane models of *S. aureus* and *E. coli* was performed to determine with which phospholipid the carboxy-terminal structure (residues 18–23) was destabilized. It was observed that the interaction of POPG from *S. aureus* with WT occurs mainly via hydrogen bonds between histidine 20 and the phospholipid polar region, generating destabilization and loss of the helical structure in that region (Figure 9A). This same residue forms a hydrogen bond with a carbonyl from POPE in the *E. coli* membrane (Figure 9B) and it was also observed that this interaction was maintained during most of the simulation without losing the helical conformation of the peptide.

## 3. Discussion

The search for new antibiotics is currently intensifying due to the increase of cases of bacterial resistance, which represents a major threat to public health worldwide [22]. Among the strategies being used, the design of antimicrobial peptides (AMPs) from sequences already reported has been pursued, with the purpose of enhancing antimicrobial activity while reducing protease sensitivity and toxicity [23]. Here, we used Alyteserin 1c as a template peptide due to its reduced cationic charge and the presence of hydrophobic residues on the polar face of its helix. Rational substitution of residues on the polar face of the helix increased the charge from +2 to +5 and subsequently increased the antimicrobial activity against Gram-positive bacteria and the hemolytic toxicity, while reducing the resistance toward staphylococcal peptidases and the activity against Gram-negative bacteria. These changes increased the selectivity toward Gram-positive bacteria and altered the three-dimensional structure of the peptide.

Throughout the broth microdilution test, it was determined that both peptides had the ability to kill bacteria despite their structural differences. It is widely known that antimicrobial peptides act primarily on the anionic membrane of microorganisms, altering the barrier function and increasing permeability [2,7,24]. There is a common feature in both peptides, a net cationic charge. This property has been widely associated with the antimicrobial activity of AMPs [25,26,27], since the surface charge density of the membrane determines the magnitude of the electrostatic attraction (Coulombica), attracting the positively charged molecules of the peptide to the negatively charged lipid membranes [28]. According to the outer surface of Gram-positive bacteria, both cationic peptides could be electrostatically attracted by anionic groups that are positioned outside the cell wall due to the presence of carboxylic groups of peptidoglycan peptides and phosphate groups of teichoic acids [29]. On the other hand, in Gram-negative bacteria, the self-promoted absorption pathway is a mechanism in which the cationic peptide displaces the divalent cations associated with lipopolysaccharides (LPS), destabilizing the macromolecular complex and facilitating internalization of the peptide towards the inner membrane [30]. Our results showed that the WT peptide exhibited greater activity against Gram-negative bacteria, while its cationic analog ΔM did this against Gram-positive bacteria, suggesting that the charge does not have a completely linear relationship with the antibacterial activity. Giangaspero et al. [25] reported that a P19 peptide analog with net charge increased to +8 showed better antimicrobial activity in yeast but reduced activity in bacteria. In another study by Jiang et al. [26], it was shown that peptides with a charge of +8 had the highest activity in Gram-negative bacteria, but reduced activity against Gram-positive bacteria in comparison to their analog with +6. To understand how the substitutions of residues decrease the antibacterial activity for Gram-negative bacteria but increase that for Gram-positive ones, we must adopt two approaches discussed below: (i) consider the external envelope of Gram-positive and Gram-negative bacteria and (ii) consider the behavior of each peptide when interacting with the membranes of both bacteria.

(i) The cell wall of Gram-positive bacteria is thicker than the wall of Gram-negative ones and can hardly be crossed by dimerized peptides. In the case of the ΔM peptide, which is more cationic than WT, the peptide chains can be repelled, avoiding dimerization and facilitating diffusion through the thick cell wall [26], while the WT peptide needs a high concentration to cross the cell wall. In contrast to Gram-positive bacteria, the antibacterial activity of the ΔM peptide in Gram-negative bacteria was reduced. Substitutions of alanines 8 and 18 for serines on the polar side of the helix could have made it difficult for the peptide to translocate through the outer membrane to reach the periplasmic space and the inner membrane surface. Permeabilization of the inner membrane is a lethal event for Gram-negative bacteria [31]. The substitution of these hydrophobic amino acids could decrease the ability for insertion into the hydrophobic core of the outer or inner membrane, as the affinity for lipids decreases. Hydrophobicity is an important parameter for the antibacterial activity of the peptide, since it controls the extent to which the peptide can be introduced into the hydrophobic core of the membrane [24]. In a previous study, it was determined that the reduction in hydrophobicity due to the absence of amino acids with large aliphatic side chains completely abolished the activity against Gram-negative and Gram-positive bacteria [25]. Other studies support the idea that hydrophobicity is more related to hemolytic activity than to antibacterial activity [32,33]. However, our results showed that hemolytic activity increased as a result of hydrophobic amino acid substitutions. This could be due to the fact that the ΔM peptide, being more cationic, is more strongly attracted by the negative charge of sialic acid, a component of the glycocalyx that forms the outer layer of erythrocytes [31].

(ii) After the peptides cross the cell wall, they reach the membrane. The helical stability of the peptides was studied by molecular dynamics using two different membrane models, *E. coli* and *S. aureus*, in order to understand the antibacterial selectivity that emerges after the replacement of the residues. It is well known that the most frequent secondary structure in AMPs is the α-helix, given the importance of its interactions with other proteins, nucleic acids, and lipids of cell membranes [34,35]. In fact, the type of phospholipid modulates the interactions with the peptide, having an impact on the stability of its structure and therefore on its activity [36]. For example, in ΔM peptide, the flexibility was higher in *E. coli* than in *S. auerus*. Flexibility is an important property that affects antimicrobial activity, normally there is greater flexibility in the central region of the peptide that reflects a hinge, which facilitates contact with the apolar region of phospholipids and thus can be inserted into the membrane [37]. The flexibility in the peptides is affected by hydrophobic interactions and intermolecular hydrogen bonds that end up altering the functionality of the peptide, for example, the binding of the peptide with phospholipid can change the flexibility and also modify its conformation [11,38]. Therefore, to understand the dynamics of hydrogen bonds in these membrane systems, the interactions with residues 18–23 of both peptides were analyzed, showing a greater number of hydrogen bonds of these residues when interacting with POPE than with POPG. The hydrogen bonds between POPE and the WT peptide are formed via lipid carbonyls. Pink et al. [39] reported that phosphate or carbonyl groups of phosphatidylethanolamine can form hydrogen bonds with other functional groups. According to our results, histidine 20 has the ability to form hydrogen bonds, especially by the imidazole group, since its participation in the formation of salt bridges and other types of noncovalent interaction has been reported [40]. This amino acid contributed both to the stability of the helical structure in the case of WT peptide in *E. coli* and of ΔM in *S. aureus*, and to the instability of the WT peptide in *S. aureus* and of ΔM in *E. coli*. These stability changes can affect the antimicrobial activity, as is the case of the WT peptide in the *S. aureus* model, in which the loss of helicity between residues 18 and 23 caused a decrease in the antibacterial effect, which similarly occurred with the ΔM peptide in *E. coli*. Tossi et al. [41] showed that destabilization of the structure led to considerable loss of antibacterial activity. These variations of hydrogen bonds depending on the type of membrane reflect the interactions with phospholipids that can alter not only the stability but also the flexibility of the peptide, interfering with its function [42]. Although both peptides retain the His20 residue, the replacement of an aliphatic chain (Ala18) by an OH group (Ser18) probably would promote the formation of hydrogen bonds between the hydroxyl of the serine side chain and the hydroxyl from cardiolipin exposed on the surface of *S. aureus* membrane, contributing with the interaction of His20. Meanwhile, methyl groups over the quaternary amine of phosphatidylethanolamine in *E. coli* would hinder the formation of hydrogen bonds with the hydroxyl of the substituted serine 18.

## 4. Materials and Methods 

### 4.1. Strains and Reagents

*S. aureus* ATCC25923, ATCC29213, ATCC43300, *L. monocytogenes* ATCCbaa751, *B. cereus* ATCC11788, *E. coli* ATCC25922, *P. aeruginosa* ATCC9027, and *S. typhimurium* ATCC14028 were obtained from the American Type Culture Collection (ATCC; Rockville, MD, USA). Mueller Hinton broth was purchased from Merck (Darmstadt, Germany).

### 4.2. Design and Synthesis of Peptides

As a template sequence for amino acid substitution, the Alyteserin-1c (WT) peptide with a net charge of +2 and PDB code 2L5R, consisting of 23 amino acid residues (GLKEIFKAGLGSLVKGIAAHVAS), was used. Four hydrophobic and/or anionic residues located on the polar face of the helix (highlighted in bold) were replaced to generate a more cationic and hydrophilic analog peptide, ΔM (GLKRIFKSGLGKLVKGISAHVAS). The synthesis and purification of Alyteserin 1c and its more cationic and hydrophilic analog were performed in accordance with the work of Cantor et al. [16]. To verify purity, a Bruker Daltonics mass spectrometer (Bruker Daltonics Inc., Billerica, USA) of the Microflex MALDI-TOF series was used.

### 4.3. Structural Modeling of Peptides

Structural models of the peptides were obtained using the I-TASSER platform (https://zhanglab.ccmb.med.umich.edu/I-TASSER/) as a tool to align the peptide sequence with the protein bank database RSCB Protein Data Bank (https://www.rcsb.org/). From the 2L5R model generated by I-TASSER and chosen for its high C-score, which represents high confidence of the structural model [43], 100 molecular models were built for the WT and ΔM peptides using MODELLER 9.14 [44]. Specifically, the models were built using the default auto-mode methods and environmental classes of MODELLER. The final models were selected according to the discrete optimized protein energy score (DOPE score). This score evaluates the energy of the models and indicates the best probable structures. The best models were evaluated through PROSA II (https://prosa.services.came.sbg.ac.at/prosa.php) and RAMPAGE (http://mordred.bioc.cam.ac.uk/~rapper/rampage.php), verifying the stereochemical quality of a protein structure through the Ramachandran plot. The models chosen had to have more than 90% amino acid residues in the favored and additional regions allowed. The structures were visualized with PyMOL (http://www.pymol.org).

### 4.4. Antimicrobial Activity

Antimicrobial assays were performed according to the Clinical and Laboratory Standards Institute, CLSI [45] with slight modifications. A bacterial culture in Mueller Hinton broth in exponential phase was diluted in Mueller Hinton broth until reaching an OD_625_ of 0.1 (approximately 1 × 10^8^ CFU/mL). Then, this culture was diluted once more by a factor of 1:200. Subsequently, 90 µL of bacterial culture was incubated with 10 µL of peptide to reach a final inoculum of approximately 5 × 10^5^ CFU/mL. The incubation time was 18–20 h in sterile 96-well plates at 37 °C. The peptides were applied at different serial dilutions (diluted in half) from 250 μM to 0.97 μM. As a negative control, phosphate-buffered saline (PBS; 138 mM NaCl, 3 mM KCl, 1.5 mM NaH_2_PO_4_, 8.1 mM Na_2_HPO_4_, pH 7.4) was used. After incubation, bacterial growth was visualized to obtain the minimum inhibitory concentration (MIC).

### 4.5. Prediction of Peptide Digestion by Staphylococcal Enzyme

The sequences of the peptides were analyzed with the PeptideCutter program of the ExPasy server (http://web.expasy.org/peptide_cutter/) to predict the sites of cleavage by staphylococcal peptidase I.

### 4.6. Hemolytic Effect

One milliliter of peripheral blood extracted from healthy young donors was washed with PBS three times by centrifuging at 3200 rpm for 5 min and discarding the supernatant. The erythrocyte button was diluted with PBS to a concentration of 4%. Subsequently, 90 μL of the final suspension of human erythrocytes with 10 μL of peptide at different concentrations in serial dilution from 125 to 3.9 μM was incubated for 1 h at 37 °C. Triton X-100 (1%) was used as a positive control and PBS as a negative control. After incubation, the solution was centrifuged at 3200 rpm and the supernatant was measured by absorbance at 540 nm on a UV 1800 spectrophotometer (Shimadzu, Japan). The percentage hemolysis was defined as follows: [(Abs Pept − Abs PBS)/(Abs Triton X100 − Abs PBS)] × 100 [2]. The minimum hemolytic concentration (MHC) is the lowest concentration at which erythrocytes are lysed. The results are expressed as the mean of three independent experiments, each performed in duplicate.

### 4.7. Construction of Gram-Negative and Gram-Positive Bacterial Membrane Models

Two membrane models, one from *E. coli* (Gram-negative) and the other from *S. aureus* (Gram-positive), were developed using CHARMM-GUI [46] and each one was developed with two types of phospholipids, in accordance with the work of Epand et al. [47]. For the Gram-negative membrane, were used POPE (1-palmitoyl-2-oleoyl-sn-glycerol-3-phosphatidylethanolamine) and POPG (1-palmitoyl-2-oleyl-sn-glycerol-3-[phospho-rac]-(1-glycerol)]), distributing 80 molecules of POPE and 20 of POPG in both outer and inner monolayers. On the other hand, the phospholipids used for the Gram-positive membrane were distributed as follows: 60 molecules of POPG and 40 molecules of PMLC1 (cardiolipin) in both outer and inner monolayers. The WT and ∆M peptides were localized in the center of each membrane (Supplementary Figure S2) in all four systems.

### 4.8. Molecular Dynamic Simulation

Molecular dynamic simulations were performed with NAMD software version 2.13 [48]. The concentration of KCl was 0.15 M as determined by the ion placing method, with water thickness of 22.5 Å and CHARMM36m as a force field [49]. The systems were adjusted by slowly heating to a temperature of 310 °K at 1 fs (femtosecond)/step for 75 ps (picoseconds) so that the conformation of the peptide is completely inserted into the membrane and to ensure that the system has no steric clashes or inappropriate geometry, relaxing the structure by energy minimization. For equilibration step 300 ps at 2 fs/step was used. Once the system is equilibrated at the desired temperature and pressure MD was ran for data collection for 10 ns. The Particle Mesh Ewald (PME) summation was applied to correct for long-range electrostatic interactions [50]. The bonds involving hydrogen atoms were restricted using the SHAKE algorithm to their minimized energy values [51], which allowed a numerical integration time step of 2 fs to be used in the simulation [50]. The helical structure has structural importance for antibacterial activity; therefore, to determine the stability of this structure during 10 ns or 10,000 ps, visual molecular dynamics (VMD) [52] and GROMACS were used [53]. In the VMD analysis, the secondary structure over the simulation time was determined using the STRIDE algorithm [54] and the number of hydrogen bonds between POPE, POPG, and residues 18 to 23 of the peptides. To work with GROMACS (version 2019.3), the .dcd format generated by NAMD was converted to .xtc using MDTRAJ [55], with the “mdconvert” command. Subsequently, the RMSD of the backbone and the radius of gyration were obtained using the gmx rms and gmx gyrate commands, respectively. To observe at which point the helical structure of the WT peptide was lost in residues 18–23 within *the S. aureus* membrane model, PYMOL [56] was used to obtain the closest links to these residues.

## 5. Conclusions

Substitutions of the amino acids in the sequence of Alyteserin 1c were implemented in order to approximate the sequence to an ideal helix, where hydrophobic residues mostly comprise one side of the helix and cationic/hydrophilic residues mostly the other side. After such substitutions, it was described how hydrophobicity, charge, and susceptibility to bacterial proteases influence the biological activity of the peptides in both bacterial and human erythrocytes. In addition, it was observed that the substitutions increased activity against Gram-positive bacteria, reducing MIC by one-third in *L. monocytogenes*. Additionally, the ΔM peptide exhibited antibacterial activity against *S. aureus* MSSA and MRSA, unlike the WT peptide, which showed no activity at the highest concentrations. On the other hand, these substitutions reduced the activity against Gram-negative bacteria. This selectivity towards Gram-positive bacteria as a result of the substitutions on the polar face of the helix was explained by a study of the peptide–membrane interaction using molecular dynamics; it was concluded that the structural stability of the peptides was associated with their antimicrobial activity. When the structure was altered after interacting with *E. coli* membranes (for ΔM) and with *S. aureus* membranes (for WT), the antimicrobial activity was reduced in each case. Finally, it was established that His20 of the peptide appears to play an essential role in said stability, as a result of the hydrogen bonds formed between it and the phospholipids in each type of membrane.

## Figures and Tables

**Table 1:** Minimum inhibitory concentration (µM) and minimum hemolytic concentration (µM) of both peptides, WT and ΔM.

Peptide	MHC	*S. aureus* ATCC25923	*S. aureus* ATCC29213	*S. aureus* ATCC43300	*L. monocytogenes* ATCCbaa751	*B. cereus* ATCC11778	*E. coli* ATCC25922	*P. aeruginosa* ATCC9027	*S. typhimurium* ATCC14028
		MIC	MIC	MIC	MIC	MIC	MIC	MIC	MIC
WT	62.5	250	ND	ND	125	ND	15.2	31.3	62.5
ΔM	31.3	62.5	250	250	62.5	125	62.5	250	125

ND = Not determined at the maximum concentration of peptide evaluated (250 µM). MIC = Minimum inhibitory concentration. MHC = Minimum hemolytic concentration.

**Table 2:** Prediction of the cleavage sites of staphylococcal peptidase I in the sequence of each peptide.

Peptide	Cut Numbers	Residue at the Cut Site
WT	1	4
ΔM	0	-

## Supplementary Materials

The following are available online at https://www.mdpi.com/2079-6382/8/4/238/s1, Figure S1: Timeline analysis of the secondary structure of the WT (A) and ∆M (B) peptides interacting with *E. coli* and *S. aureus* systems. Figure S2: Peptide placed inside the membrane model.

## References

[1] Hiemstra P.S., Zaat S.A.J. (2013). Antimicrobial Peptides and Innate Immunity.

[2] Oñate-Garzón J., Manrique-Moreno M., Trier S., Leidy C., Torres R., Patiño E. (2017). Antimicrobial activity and interactions of cationic peptides derived from Galleria mellonella cecropin D-like peptide with model membranes. J. Antibiot..

[3] Li W., Tailhades J., O’Brien-Simpson N.M., Separovic F., Otvos L., Hossain M.A., Wade J.D. (2014). Proline-rich antimicrobial peptides: Potential therapeutics against antibiotic-resistant bacteria. Amino Acids.

[4] Fjell C.D., Hiss J.A., Hancock R.E., Schneider G. (2012). Designing antimicrobial peptides: Form follows function. Nat. Rev. Drug Discov..

[5] Mangoni M.L., Mcdermott A.M., Zasloff M. (2016). Antimicrobial peptides and wound healing: Biological and therapeutic considerations. Exp. Dermatol..

[6] Strempel N., Strehmel J., Overhage J. (2014). Potential Application of Antimicrobial Peptides in the Treatment of Bacterial Biofilm Infections. Curr. Pharm. Des..

[7] Lee T.H., Hall K.N., Aguilar M.I. (2016). Antimicrobial Peptide Structure and Mechanism of Action: A Focus on the Role of Membrane Structure. Curr. Top. Med. Chem..

[8] Oñate-Garzón J., Ausili A., Manrique-Moreno M., Torrecillas A., Aranda F.J., Patiño E., Gomez-Fernández J.C. (2017). The increase in positively charged residues in cecropin D-like Galleria mellonella favors its interaction with membrane models that imitate bacterial membranes. Arch. Biochem. Biophys..

[9] Subasinghage A.P., O’Flynn D., Conlon J.M., Hewage C.M. (2011). Conformational and membrane interaction studies of the antimicrobial peptide alyteserin-1c and its analogue [E4K]alyteserin-1c. Biochim. Biophys. Acta.

[10] Conlon J.M., Demandt A., Nielsen P.F., Leprince J., Vaudry H., Woodhams D.C. (2009). The alyteserins: Two families of antimicrobial peptides from the skin secretions of the midwife toad Alytes obstetricans (Alytidae). Peptides.

[11] Aragón-Muriel A., Ausili A., Sánchez K., Rojas A., Oscar E., Londoño Mosquera J., Polo-Cerón D., Oñate-Garzón J. (2019). Studies on the Interaction of Alyteserin 1c Peptide and Its Cationic Analogue with Model Membranes Imitating Mammalian and Bacterial Membranes. Biomolecules.

[12] Berglund N.A., Piggot T.J., Jefferies D., Sessions R.B., Bond P.J., Khalid S. (2015). Interaction of the antimicrobial peptide polymyxin B1 with both membranes of E. coli: A molecular dynamics study. PLoS Comput. Biol..

[13] Ulmschneider J.P., Ulmschneider M.B. (2018). Molecular Dynamics Simulations Are Redefining Our View of Peptides Interacting with Biological Membranes. Acc. Chem. Res..

[14] Velasco-Bolom J.L., Corzo G., Garduño-Juárez R. (2018). Molecular dynamics simulation of the membrane binding and disruption mechanisms by antimicrobial scorpion venom-derived peptides. J. Biomol. Struct. Dyn..

[15] Bordo D., Argos P. (1991). Suggestions for “safe” residue substitutions in site-directed mutagenesis. J. Mol. Biol..

[16] Cantor S., Vargas L., Rojas O.E.A., Yarce C.J., Salamanca C.H., Oñate-Garzón J. (2019). Evaluation of the antimicrobial activity of cationic peptides loaded in surface-modified nanoliposomes against foodborne bacteria. Int. J. Mol. Sci..

[17] Würz J.M., Güntert P. (2017). Peak picking multidimensional NMR spectra with the contour geometry based algorithm CYPICK. J. Biomol. NMR.

[18] Wiederstein M., Sippl M.J. (2007). ProSA-web: Interactive web service for the recognition of errors in three-dimensional structures of proteins. Nucleic Acids Res..

[19] Beg M.A., Shivangi, Thakur S.C., Meena L.S. (2018). Structural Prediction and Mutational Analysis of Rv3906c Gene of Mycobacterium tuberculosis H37Rv to Determine Its Essentiality in Survival. Adv. Bioinform..

[20] Rhodes G. (2006). Other Diffraction Methods. Crystallogr. Made Cryst. Clear.

[21] Lobanov M.I., Bogatyreva N.S., Galzitskaia O.V. (2008). Radius of gyration is indicator of compactness of protein structure. Mol. Biol. (Mosk)..

[22] Waghu F.H., Joseph S., Ghawali S., Martis E.A., Madan T., Venkatesh K.V., Idicula-Thomas S. (2018). Designing antibacterial peptides with enhanced killing kinetics. Front. Microbiol..

[23] Vishnepolsky B., Zaalishvili G., Karapetian M., Nasrashvili T., Kuljanishvili N., Gabrielian A., Rosenthal A., Hurt D.E., Tartakovsky M., Grigolava M. (2019). De Novo Design and In Vitro Testing of Antimicrobial Peptides against Gram-Negative Bacteria. Pharmaceuticals.

[24] Teixeira V., Feio M.J., Bastos M. (2012). Role of lipids in the interaction of antimicrobial peptides with membranes. Prog. Lipid Res..

[25] Giangaspero A., Sandri L., Tossi A. (2001). Amphipathic alpha helical antimicrobial peptides. Eur. J. Biochem..

[26] Jiang Z., Vasil A.I., Hale J.D., Hancock R.E., Vasil M.L., Hodges R.S. (2008). Effects of net charge and the number of positively charged residues on the biological activity of amphipathic alpha-helical cationic antimicrobial peptides. Biopolymers.

[27] Oñate-Garzón J.F., Manrique-Moreno M., Patiño González E. (2017). Actividad antimicrobiana de péptidos catiónicos diseñados a partir de un péptido neutro. Acta Biológica Colomb..

[28] Abraham T., Lewis R.N., Hodges R.S., McElhaney R.N. (2005). Isothermal titration calorimetry studies of the binding of a rationally designed analogue of the antimicrobial peptide gramicidin s to phospholipid bilayer membranes. Biochemistry.

[29] Sonnenfeld E.M., Beveridge T.J., Koch A.L., Doyle R.J. (1985). Asymmetric distribution of charge on the cell wall of Bacillus subtilis. J. Bacteriol..

[30] Hancock R.E. (1997). Peptide antibiotics. Lancet.

[31] Papo N., Shai Y. (2003). Can we predict biological activity of antimicrobial peptides from their interactions with model phospholipid membranes?. Peptides.

[32] Chen Y., Guarnieri M.T., Vasil A.I., Vasil M.L., Mant C.T., Hodges R.S. (2007). Role of peptide hydrophobicity in the mechanism of action of alpha-helical antimicrobial peptides. Antimicrob. Agents Chemother..

[33] Chou H.T., Kuo T.Y., Chiang J.C., Pei M.J., Yang W.T., Yu H.C., Lin S.B., Chen W.J. (2008). Design and synthesis of cationic antimicrobial peptides with improved activity and selectivity against Vibrio spp.. Int. J. Antimicrob. Agents.

[34] Zelezetsky I., Tossi A. (2006). Alpha-helical antimicrobial peptides—Using a sequence template to guide structure-activity relationship studies. Biochim. Biophys. Acta.

[35] Yakimov A.P., Afanaseva A.S., Khodorkovskiy M.A., Petukhov M.G. (2016). Design of Stable a-Helical Peptides and Thermostable Proteins in Biotechnology and Biomedicine. Acta Naturae.

[36] Bondar A.N., White S.H. (2012). Hydrogen bond dynamics in membrane protein function. Biochim. Biophys. Acta Biomembr..

[37] Liu L., Fang Y., Wu J. (2013). Flexibility is a mechanical determinant of antimicrobial activity for amphipathic cationic α-helical antimicrobial peptides. Biochim. Biophys. Acta Biomembr..

[38] Mamonova T., Hespenheide B., Straub R., Thorpe M.F., Kurnikova M. (2005). Protein flexibility using constraints from molecular dynamics simulations. Proc. Phys. Biol..

[39] Pink D.A., Belaya M., Levadny V., Quinn B. (1997). A model of polar group statics in lipid bilayers and monolayers. Langmuir.

[40] Krishna Deepak R.N.V., Sankararamakrishnan R. (2016). N-H···N Hydrogen Bonds Involving Histidine Imidazole Nitrogen Atoms: A New Structural Role for Histidine Residues in Proteins. Biochemistry.

[41] Tossi A., Scocchi M., Skerlavaj B., Gennaro R. (1994). Identification and characterization of a primary antibacterial domain in CAP18, a lipopolysaccharide binding protein from rabbit leukocytes. FEBS Lett..

[42] Teilum K., Olsen J.G., Kragelund B.B. (2011). Protein stability, flexibility and function. Biochim. Biophys. Acta Proteins Proteom..

[43] Roy A., Kucukural A., Zhang Y. (2010). I-TASSER: A unified platform for automated protein structure and function prediction. Nat. Protoc..

[44] Webb B., Sali A. (2016). Comparative protein structure modeling using MODELLER. Curr. Protoc. Bioinforma..

[45] Clinical and Laboratory Standards Institute (CLSI) (2015). Methods for Dilution Antimicrobial Susceptibility Tests for Bacteria That Grow Aerobically.

[46] Jo S., Kim T., Iyer V.G., Im W. (2008). CHARMM-GUI: A web-based graphical user interface for CHARMM. J. Comput. Chem..

[47] Epand R.F., Savage P.B., Epand R.M. (2007). Bacterial lipid composition and the antimicrobial efficacy of cationic steroid compounds (Ceragenins). Biochim. Biophys. Acta Biomembr..

[48] Phillips J.C., Braun R., Wang W., Gumbart J., Tajkhorshid E., Villa E., Chipot C., Skeel R.D., Kalé L., Schulten K. (2005). Scalable molecular dynamics with NAMD. J. Comput. Chem..

[49] Huang J., Rauscher S., Nawrocki G., Ran T., Feig M., De Groot B.L., Grubmüller H., MacKerell A.D. (2016). CHARMM36m: An improved force field for folded and intrinsically disordered proteins. Nat. Methods.

[50] Legge F.S., Treutlein H., Howlett G.J., Yarovsky I. (2007). Molecular dynamics simulations of a fibrillogenic peptide derived from apolipoprotein C-II. Biophys. Chem..

[51] Ciccotti G., Ryckaert J.P. (1986). Molecular dynamics simulation of rigid molecules. Comput. Phys. Reports.

[52] Humphrey W., Dalke A., Schulten K. (1996). VMD: Visual molecular dynamics. J. Mol. Graph..

[53] Berendsen H.J.C., van der Spoel D., van Drunen R. (1995). GROMACS: A message-passing parallel molecular dynamics implementation. Comput. Phys. Commun..

[54] Frishman D., Argos P. (1995). Knowledge-based protein secondary structure assignment. Proteins Struct. Funct. Bioinform..

[55] McGibbon R.T., Beauchamp K.A., Harrigan M.P., Klein C., Swails J.M., Hernández C.X., Schwantes C.R., Wang L.P., Lane T.J., Pande V.S. (2015). MDTraj: A Modern Open Library for the Analysis of Molecular Dynamics Trajectories. Biophys. J..

[56] DeLano W.L. (2002). PyMOL: An open-source molecular graphics tool. Ccp4 Newslett. Protein Crystallogr..

